# Manual of Physiology

**Published:** 1849-11

**Authors:** 


Art. III.—*Manual of Physiology.* By W. Senhouse Kirkes, M. D. Assisted by James Paget, Lecturer on General Anatomy and Physiology, at St. Bartholomew's Hospital, with 118 illustrations on wood. Philadelphia: Lea & Blanchard. 12mo. pp. 548. 1849.

In our last number, we presented the views of our authors respecting the functions of Respiration and Animal Heat. The next subject discussed in their interesting work is Digestion, their account of which is full and satisfactory.

Digestion, by some physiologists, is held to be a purely *vital* process, with which chemical laws have nothing to do, and which chemistry can neither imitate nor explain. Others, on the contrary, regard its phenomena as belonging to the category for which chemistry alone is able to account. They look upon digestion as a process of *catalysis*, or of fermentation, in which, by simple contact, or, at most, by contact with matter in a state of change, the food is broken down in its constitution, dissolved, converted into chyme, and fitted to enter the circulation for the nourishment of the system. There is reason to believe that both these extreme parties are in error, and that in this question, as in so many similar cases, we are safer on middle ground. The facts thus far brought to light do not warrant us in assuming that digestion is foreign in all its stages from operations which we refer to chemical affinity, much less that it is in antagonism with those forces. The opinion that the vital principle, as exerted in digestion, is at war with chemical affinity, has no foundation in nature. But, at the same time, since digestion is accomplished within living beings, and by the agency of a fluid secreted by the stomach, we do not see how the conclusion can be resisted that the process is a vital one—a process of *vital chemistry.*

Thus, it takes place out of the living body ; it goes on after life becomes extinct, as when the stomach has been found digested in instances of sudden death ; it may be performed by an artificial digestive fluid, and therefore is not wholly vital. But the fluid by which it is effected out of the stomach, by which the stomach itself is dissolved after death, is a fluid formed under the influence of the vital principle ; and the artificial digestive fluid must have a product of that principle in order to fulfil the object. Such a fluid is made by macerating in water portions of the mucous membrane of the stomach of a pig or of some other animal, to which a few drops of hydrochloric acid have been added. Without the animal secretion it is inefficient. The process may be imitated, but the agency of life seems as indispensable out of the living body as within it. When once the gastric fluid is secreted, digestion may be regarded as independent of life, and therefore, in the language of Dr. Kirkes, "as essentially a chemical process"; but up to this period all depends upon vitality, and hence in this stage it must be held a vital process. The forces by which it is accomplished are so mixed up that it is impossible to say where the influence of one ceases and the others begin to operate.

The digestive power of the gastric fluid taken from the stomach and kept at a temperature of 100°, is seen in its softening and partially or completely converting into chyme the various articles of food subjected to its action. But this is only done when pepsine and the acid of the stomach both are present. By neither can it be performed alone, and, when mixed, if the pepsine be decomposed or the acid neutralized, the digestive property of the fluid is rendered null. The rationale of the process, as attempted to be given by Dr. K., in the following extract is, perhaps, as near the truth as any that can be offered in the present state of our knowledge on the subject.

The nature of the action by which the mucous membrane of the stomach, and its secretion, work these changes in organic matter, is exceedingly obscure. The action of the pepsine may be compared with that of a ferment, which, at the same time that it undergoes change itself, induces certain changes also in the organic matters with which it is in contact. Or its mode of action may belong to that class of chemical processes termed "catalytic," in which a substance excites, by its mere presence, and without itself undergoing change as ordinary ferments do, some chemical action in the substances with which it is in contact. So, for example, spongy platinum placed in a mixture, however voluminous, of oxygen and hydrogen, makes them combine to form water; and diastase makes the starch in grains undergo transformation, and sugar is produced. And that pepsine acts in some such manner appears probable from the very minute quantity capable of exerting the peculiar digestive action on a large quantity of food, and apparently with little diminution in its active power. The process differs from ordinary fermentation in being unattended with the formation of carbonic acid, in not requiring the presence of oxygen, and in being unaccompanied by the production of new quantities of the active principle, or ferment. It agrees with the processes of both fermentation and organic catalysis in that whatever alters the composition of the pepsine (such as heat above 110°, strong alcohol, or strong acids), destroys the digestive power of the fluid.

Alimentary substances are divided into two classes—the *nutritive*, or nitrogenized, and the *respiratory*, or non-nitrogenous. Matters destitute of nitrogen are incapable of taking any part in the formation and repair of the animal tissues. Food is also divided into classes having reference to its origin; but in animal and vegetable substances the nutritive principles are the same. It is the same fibrine, albumen, and caseine that go to build up the frame and repair its waste, whether the articles of food be derived from the animal or vegetable kingdom. The food of the carnivora is identical in composition with their own tissues, and consequently little more seems requisite to their nutrition than that it should be dissolved and conveyed into the blood in a condition capable of being re-organized. The food of the herbivora seems widely different, but essentially the parts for which they consume it are identical with the muscular flesh which forms the aliment of carnivorous animals. The process of digestion with them is more tedious, requiring more complicated apparatus, but in the last analysis it consists, as in the carnivora, in the solution and assimilation of the albumen or its cognate elements.

While plants are nourished by water, carbonic acid, and ammonia, it is only upon organic substances that animals can subsist; and as with the three compounds just mentioned other mineral matters must be furnished to plants to perfect their development, so even organic compounds are not sufficient for the nutrition of animals unless they are supplied in their natural state. Animals, as well as vegetables, require earthy materials in their aliment. Men are attacked with scurvy if they are too long deprived of vegetable food; and it is necessary to have the vegetables either raw, or so cooked as not to remove from them their saline constituents. The plastic elements, fibrine, albumen, and caseine are, therefore, not sufficient, in themselves, and can only support life for a brief period.

Moreover, health cannot be maintained by any number of substances derived exclusively from one of the three groups of alimentary principles. A mixture of nitrogenous and non-nitrogenous substances, together with the inorganic principles which are severally contained in them, is essential to the well-being, and, generally, even to the existence of an animal. The truth of this is demonstrated by experiments performed for the purpose, and is illustrated by the composition of the food prepared by nature as the exclusive source of nourishment to the young of mammalia, namely milk. In milk, the albuminous group of aliments is represented by the caseine, the oleaginous by the butter, the aqueous by the water, the saccharine by the sugar of milk. Milk, likewise, contains phosphate of lime, alkaline and other salts, and a trace of iron: so that it may be briefly said to include all the substances which the tissues of the growing animal need for their nutrition, and which are required for the production of animal heat. The yelk and albumen of eggs are in the same relation, as food for the embryos of oviparous animals, as milk is to the young of mammalia, and afford another example of mixed food being provided as the most perfect for nutrition.

Magendie has performed numerous experiments illustrating this principle. He fed dogs exclusively on sugar and distilled water; and although for a week or more they were active and took their food as usual, in the course of the second week they began to lose flesh, and in spite of a good appetite became more and more emaciated, and finally died from the thirty-first to the thirty-fourth day. On dissection their bodies exhibited all the signs of death from starvation.

Besides the comminution of the food, which is effected in the mouth, other changes take place in that cavity.—The saliva exerts a chemical action upon some of the elements of food, converting starch into dextrine and grape sugar. This would seem to be one of the objects of insalivation, for the gastric juice tends rather to retard than to favor the transformation of the starch. From experiments performed by Magendie and Bernard, it appears that many substances besides saliva may induce these changes in starch, such as pieces of any of the mucous membranes; so that the property cannot be assigned to any peculiar organic principle in this secretion. Nor indeed is any such property required in the saliva of carnivorous animals, since their food is destitute of farinaceous substances. It is not known that saliva exerts any kind of digestive power over animal matters, and we are therefore uncertain whether it subserves any other purpose in the carnivora than that of lubricating their food.

Swallowing is in part an involuntary act. We carry back the morsel of food to a particular point by muscles which are under the control of the will; but when it has reached a certain part of the tongue's surface, muscular contraction takes place without our being able to prevent it.

When we appear to swallow voluntarily, we only convey, through the first act of deglutition, a portion of food or saliva beyond the anterior arch of the palate; thus, the substance acts as a stimulus which in accordance with the laws of reflex movements hereafter to be described, is carried by the sensitive nerves to the medulla oblongata, where it is reflected to the motor nerves, and an involuntary adapted action of the muscles of the palate and pharynx ensues. The third act of deglutition takes place in the esophagus, the muscular fibres of which are entirely beyond the influence of the will.

The mechanism of deglutition is exceedingly beautiful. There are two passages besides the right one, into which the food might make its way, and induce distressing and even fatal consequences. It must pass in its course to the esophagus by the posterior orifice of the nose, and over the rima glottidis of the larynx, without touching them; and so exquisitely have the motions been adjusted that it rarely errs from the right course. As the morsel is carried backwards by the tongue, the larynx is raised and carried forwards under the tongue, and the rima glottidis is closed by the epiglottis; but even where the latter is destroyed, the closure of the glottis is secured by the simultaneous contraction of its own muscles. "At the same time, the approximation of the sides of the posterior palatine arch, which move quickly inwards like side-curtains, closes the passage into the upper part of the pharynx and the posterior nares, and forms an inclined plane, along the under surface of which the morsel descends; when the pharynx, raised up to receive it, in its turn contracts, and forces it onwards into the esophagus." Its passage through the esophagus is wholly independent of the will, the successive contractions of the tube resulting from the contact of the food with its different parts as it descends.

The gastric juice is described by nearly every experimenter as acid in its character, but the different writers are very far from being agreed as to the nature of the acid upon which digestion depends. Dr. Dunglison found in the stomach of St. Martin both acetic and hydrochloric acids; and Dr. Prout has satisfied himself of the existence of the latter acid in the gastric fluid of the rabbit, horse, calf, hare, and dog. But doubts have been created by the more recent experiments of Blondlot, Thompson, Lehmann, Bernard, and Barreswill, as to the existence of free hydrochloric, or indeed any other volatile, acid in the fluid of the healthy stomach. Dr. Kirkes relates that—

Having obtained large quantities of pure gastric fluid from the stomach of a dog, and carefully distilled portions of it on the sand bath, Blondlot found not the slightest trace of acidity in the product of the distillation; but the residue in the retort was intensely acid, and became more so the more it was concentrated by continuing the distillation. The non-existence of both hydrochloric and acetic acids seeming to be thus demonstrated, Blondlot was led to believe the acidity of the gastric fluid depends on an acid phosphate of lime. For he observed no effervescence on the addition of carbonate of lime to the acid gastric fluid; neither when carbonate of lime was placed in the gastric fluid, was the fluid neutralized, or the carbonate dissolved. By further investigation he demonstrated the existence of a super-phosphate of lime in the gastric fluid. But he seems to have been in error in attributing the whole of the acidity of the gastric fluid to this salt: for MM. Bernard and Barreswill have found that if the gastric fluid be sufficiently concentrated by evaporation, distinct effervescence occurs on the addition of carbonate of lime; proving beyond doubt the presence of some free acid, which they as well as Dr. R. D. Thompson and Lehmann, consider to be the lactic, an opinion to which Liebig also gives his sanction. MM. Melsens and Dumas have also proved the existence of a free acid by the gradual solution of portions of carbonate of lime placed in gastric fluid. But since, even after long contact, the carbonate of lime does not completely neutralize the acid of the gastric fluid, it is most probable that there is, together with a free acid, some acid phosphate of lime, as maintained by M. Blondlot.

Dr. Kirkes suggests, as an explanation of these discrepancies of various observers, "that the source of the acidity of the gastric fluid may vary in different animals, or at different times in the same animal;" and that hydrochloric acid may have been found in the human stomach by Dunglison and Prout, while lactic acid was found in the stomach of the pig and of the dog, by other experimentalists. The conclusion from the whole seems to be, as expressed by him, "that the digestive properties of the gastric fluid require only the existence of a certain degree of acidity, which is equally effective whatever be the acid employed, provided this acid does not decompose the active animal principle of the digestive fluid."

The change effected in the alimentary principles by the gastric secretion, whatever be the mode of it, is essential to their assimilation, as is shown by the late experiments of Bernard and Barreswill, who always found albumen in the urine a few hours after they had injected it, dissolved in water, into the jugular veins of dogs, but did not detect it if, previous to injection, it was mixed with the gastric fluid.

The various elements of food are variously affected by the fluids of the stomach.

The *saccharine*, including the *amylaceous* principles are at first, probably, only mechanically separated from the vegetable substances within which they are contained, by the action of the gastric fluid. The soluble portions, viz: sugar, gum, and pectine are probably at once absorbed. The insoluble ones, viz: starch and lignine (or some parts of it) are rendered soluble and capable of absorption, by being converted into dextrine and grape-sugar. It is probable that this change is carried on to some extent in the stomach; for many experiments, including those of Dr. Percy, show that starch is absorbed from the stomach, being, of course, previously rendered soluble. But the change is continued in the intestinal canal, perhaps, as will be shown, under the influence of the secretion of the pancreas. And, further, respecting the action of the stomach in the digestion of starch, it may be doubted whether the human stomach has any power over it in the raw state; for both by man and carnivora, when starch has been taken raw, as in corn and rice, large quantities of the granules are passed unaltered with the excrements. Cooking, by expanding or bursting the envelopes of the granules, renders their interior more amenable to the action of the digestive organs; and the abundant nutriment furnished by bread, and the large proportion that is absorbed of the weight of it consumed, afford proof of the completeness of their power to make its starch soluble and prepare it for absorption.

The agency by which the *oleaginous* principles are prepared for admission into the economy was not understood when this work was written; but a conjecture as to its nature was thrown out by Dr. Kirkes, the truth of which more recent researches have established. Speaking of the part performed by the *pancreas* in digestion, he says:

Perhaps it may assist in the digestion of fat, or in rendering it fit for absorption; for numerous cases are recorded in which, the pancreatic duct being obstructed so that the secretion could not be discharged, fatty or oily matter was abundantly discharged from the intestines. In nearly all these cases, indeed, the liver was coincidently diseased, and the change or absence of the bile might appear to contribute to the result: but in at least one the liver was healthy, and there appeared nothing but the absence of the pancreatic fluid from the intestines to which the excretion or non-absorption of fatty matter could be ascribed.

The reader does not need to be told that since the publication of this volume, M. Bernard has shown, by a series of most satisfactory experiments, that the function of the pancreas is precisely the one attributed to it by Dr. Kirkes. Dr. K., by an ingenious method of reasoning, had arrived at the true conclusion, which only wanted for its confirmation the simple experiments since performed by M. Bernard.

The sensation which induces to the taking of food, or *hunger*, is referred by the mind, and has generally been referred by physiologists, to the stomach; but there is good reason to believe that it does not entirely depend upon the state of that organ. For it has been found to be allayed by the introduction of food not only into the stomach, but into the blood through other channels; and it has been further ascertained that the division of both pneumogastric nerves, by which chiefly the mind is cognizant of the condition of the stomach, does not appease the cravings of hunger. Still the stomach has a share in this sensation, for the introduction into it of non-alimentary substances suspends the appetite for a time. And we may, therefore, conclude that the feeling of hunger depends upon the condition of the general system, but more especially upon the stomach, the nerves of which, we may suppose, are more affected by the state of the insufficiently replenished blood than those of other organs are.

In like manner, the *sensation of thirst* is referred to the fauces, although, as in hunger, " this is merely the local declaration of a general condition existing in the system." The truth of this position is proved by the fact that washing the dry fauces with liquids affords only temporary relief, while thirst is effectually allayed by water introduced into the circulation either through the stomach, the skin, the intestines, or directly by injection into the bloodvessels.

The sensation of thirst is perceived most naturally whenever there is a disproportionately small quantity of water in the blood; as well, therefore, when water has been abstracted from the blood, as when saline, or any solid matters have been abundantly added to it. We can express the fact (even if it be not an explanation of it,) by saying that the nerves of the mouth and fauces, through which the sense of thirst is chiefly derived, are more sensitive to this condition of the blood than other nerves are. And the cases of hunger and thirst are not the only ones in which the mind derives, from certain organs, a peculiar predominant sensation of some condition affecting the whole body. Thus, the sensation of the "necessity of breathing," is referred especially to the lungs; but, as Volkmann's experiments show, it depends on the condition of the blood which circulates everywhere, and is felt even after the lungs of animals are removed; for they continue, even then, to gasp and manifest the sensation of want of breath. So, perhaps, it may be added, the disordered blood of fever, and other affections of the blood, circulates everywhere, but produces peculiar sensations in only certain parts. And, as with respiration, when the lungs are removed, the mind may still feel the body's want of breath, so in hunger and thirst, even when the stomach has been filled with innutritious substances, or the pneumogastric nerves have been divided, and the mouth and fauces have been kept moist, the mind is still aware, by the more obscure sensations in other parts, of the whole body's need of food and water.

The influence of the nervous system upon digestion is forcibly shown by the effect of passion in arresting the process. It is well known that it may be completely suspended by a fit of anger, and nothing is better understood than the tendency of the depressing passions to induce indigestion. A paroxysm of rage just after a hearty meal has been known to lead to convulsions, and the food ejected from the stomach by vomiting twelve hours after it was taken, was found unchanged. The division of the pneumogastric nerves produces the same effect, as has been shown by M. Bernard, who watched the act of digestion in dogs through fistulous openings in their stomachs. On the instant of severing the nerves digestion appeared to cease, and the mucous membrane of the stomach, which before was turgid with blood, became pale and no longer secreted the gastric juice. It is proper to remark, however, that while in other experiments, as in those of M. Bernard, the division of the pneumogastric nerves always temporarily puts a stop to digestion and often caused death from inanition, still the stomach finally regained its function after the operation, and the processes of digestion and nutrition went on as well as before it was performed.

It has been asserted that the section of these nerves prevents the absorption of poisons by the stomach; but in the experiments performed by Wernscheidt under the direction of Müller, no difference was observed in the action of narcotic poisons whether the nerves had been divided or not. An interesting exception, however, is presented in certain cases; for, as remarked by Dr. Kirkes,

It appears that such poisons as are capable of being rendered inert by the action of the gastric fluid, may, if taken into the stomach shortly after division of both pneumogastric nerves, produce their poisonous effects, in consequence, apparently, of the temporary suspension of the secretion of gastric fluid. Thus, in one of his experiments, M. Bernard gave to each of two dogs, in one of which he had divided the pneumogastric nerves, a dose of emulsine, and half an hour afterwards a dose of amygdaline, substances which are innocent alone, but when mixed produce hydrocyanic acid. The dog, whose nerves were cut, died in a quarter of an hour, the substances being absorbed unaltered and mixing in the blood; in the other, the emulsine was decomposed by the gastric fluid before the amygdaline was administered; therefore hydrocyanic acid was not formed in the blood, and the dog survived.

In the intestines other important changes are effected in the food. The pancreas pours its secretion into the duodenum to make an emulsion of the fatty particles; and in some animals, as the solidungula, rodentia, and ruminantia, the cecum is greatly extended, apparently for the purpose of perfecting the digestive process commenced in the stomach. Even in animals of the same species the length of the large intestines is found to vary with the food, the ostrich inhabiting regions which abound in vegetation, and the ostrich of the desert conforming, in this respect, to the carnivora on the one hand, and to the herbivora on the other. The cecum is said to be absent in those classes of animals which hybernate, but the relation of this bowel to that peculiar faculty is not understood.

The liver is intimately associated with the stomach and intestines in the assimilative function, and there is much that is peculiar and interesting in its office and organization. The blood which the portal vein conveys to the liver contains portions of the soluble parts of the food.—This blood differs from the venous blood in other parts of the body in having a less specific gravity; in being darker and insusceptible of acquiring a red hue from the contact of salt, oxygen, or atmospheric air; it contains only about half the ordinary quantity of fibrine, which is in so imperfect a condition that when it coagulates the clot forms slowly and is not so firm as in other blood; it also contains less albumen, but more red globules, and nearly twice the usual amount of fatty matter. These striking characteristics, which we simply quote from the work before us, refer to the composition of the portal blood itself, and not to the various extraneous substances which frequently mingle with it. According to Bouchardat and Sandras, when herbivorous animals have been fed on farinaceous substances the blood of the portal vein contains more dextrine and grape-sugar than that of any other bloodvessel. Trommer, when grape-sugar had been fed to animals, found that substance in their portal, but not in their hepatic, veins; and Blondlot and Chossat remarked that the administration of sugar and other non-nitrogenous articles of food, materially increases the quantity of bile secreted. And, as Dr. K. continues—

These things make it probable that the food, especially the saccharine and amylaceous principles, being absorbed from the intestines by the bloodvessels and conveyed through the branches of the portal vein in the liver, contributes, with the elements of the portal blood, to supply the materials out of which the bile is formed.

The purposes served by the secretion of bile are of two principal kinds, namely, *excrementitious* and *digestive.*

As an excrementitious substance, the bile is destined especially for the preparation of portions of carbon and hydrogen in order that they may be removed from the blood: and its adaptation to this purpose is well illustrated by the peculiarities attending its secretion and disposal in the fetus. During intra-uterine life, the lungs and the intestinal canal are almost inactive: there is no respiration of open air or digestion of food; these are unnecessary, because of the supply of well-elaborated nutriment received by the vessels of the fetus at the placenta. The liver, during the same time, is proportionably larger than it is after birth, and the secretion of the bile is active, although there is no food in the intestinal canal upon wnich it can exercise any digestive property. At birth, the intestinal canal is full of thick bile, mixed with intestinal secretion; for the *meconium*, or feces of the fetus, is shown, by the analysis of Simon and of Frerich, to contain all the essential principles of bile. In the fetus, therefore, the main purpose of the secretion of bile must be the purification of the blood by *direct* excretion, *i. e.* by separation from the blood, and ejection from the body without furthur change. Probably, all the bile secreted in fetal life is incorporated in the meconium, and with it discharged; and thus the liver may be said to discharge a function in some sense vicarious of that of the lungs. For in the fetus, nearly all the blood coming from the placenta passes through the liver previous to its distribution to the several organs of the body; and the abstraction of carbon, hydrogen, and other elements of bile will purify it, as in extra-uterine life the separation of carbonic acid and water at the lungs does.

This evident disposal of the fetal bile by excretion makes it highly probable that the bile in extra-uterine life is also, at least for the most part, destined to be discharged as excrement. But the analysis of the feces of both children and adults shows that (except when rapidly discharged in purgation) they contain very little of the bile secreted, probably not more than one-sixteenth part of its weight, and that this portion includes only its coloring and some of its fatty matters, but none of its essential principle, the biline. All the biline is again absorbed from the intestines into the blood. But the elementary composition of biline shows such a preponderance of carbon and hydrogen that it cannot be appropriated to the nutrition of the tissues; therefore, it may be presumed that, after absorption, the carbon and hydrogen of the biline combining with oxygen are excreted in carbonic acid and water. The destination of the bile is, on this theory, essentially the same in both fetal and extra-uterine life; only, in the former, it is *directly* excreted, in the latter *indirectly*, being, before final ejection, modified in its absorption from the intestines and mingled with the blood.

The change from the direct to the indirect mode of excretion of the bile may, with much probability, be connected with a purpose in relation to the development of heat. The temperature of the fetus is maintained by that of the parent, and needs no source of heat within the body of the fetus itself; but, in extra-uterine life, there is (as one may say) a waste of material for heat when any excretion is discharged unoxydised : the carbon and hydrogen of the biline, therefore, instead of being ejected in the feces, are re-absorbed, in order that they may be combined with oxygen, and that in the combination heat may be generated.

That ejection is the final destination of the bile, and that whatever other purposes it may serve are not essential to the maintenance of life, appear from facts mentioned by Blondlot. He found that dogs may live in health for at least several months even though the bile is prevented from passing into the intestines by removing a portion of the common bile-duct, provided all the bile that is secreted can be discharged from the body by keeping open a fistulous communication between the skin and the gall-bladder. It must not, however, be thought indifferent whether the bile be re-absorbed or not, provided it be ejected; for, in experiments similar to those of Blondlot, Schwann found that the animals always died with the signs of inanition; such signs, it may be supposed, as would be produced by the deficiency of carbon and hydrogen in the blood.

Here, for the want of space, we are compelled to close the present article. We shall have to crave the patience of our readers while we devote a few pages more to this work in a future number.

